# The Potential Role of RP105 in Regulation of Inflammation and Osteoclastogenesis During Inflammatory Diseases

**DOI:** 10.3389/fcell.2021.713254

**Published:** 2021-08-03

**Authors:** Zhou Fan, Janak L. Pathak, Linhu Ge

**Affiliations:** ^1^Guangzhou Key Laboratory of Basic and Applied Research of Oral Regenerative Medicine, Affiliated Stomatology Hospital of Guangzhou Medical University, Guangzhou, China; ^2^Institute of Oral Disease, Guangzhou Medical University, Guangzhou, China

**Keywords:** osteoclasts, Toll-like receptors, liposaccharides, radioprotective 105kDa protein, osteoporosis

## Abstract

Inflammatory diseases have a negative impact on bone homeostasis via exacerbated local and systemic inflammation. Bone resorbing osteoclasts are mainly derived from hematopoietic precursors and bone marrow monocytes. Induced osteoclastogenesis during inflammation, autoimmunity, metabolic diseases, and cancers is associated with bone loss and osteoporosis. Proinflammatory cytokines, pathogen-associated molecular patterns, or endogenous pathogenic factors induce osteoclastogenic differentiation by binding to the Toll-like receptor (TLR) family expressed on surface of osteoclast precursors. As a non-canonical member of the TLRs, radioprotective 105 kDa (RP105 or CD180) and its ligand, myeloid differentiation protein 1 (MD1), are involved in several bone metabolic disorders. Reports from literature had demonstrated RP105 as an important activator of B cells, bone marrow monocytes, and macrophages, which regulates inflammatory cytokines release from immune cells. Reports from literature had shown the association between RP105 and other TLRs, and the downstream signaling mechanisms of RP105 with different “signaling-competent” partners in immune cells during different disease conditions. This review is focused to summarize: (1) the role of RP105 on immune cells’ function and inflammation regulation (2) the potential regulatory roles of RP105 in different disease-mediated osteoclast activation and the underlying mechanisms, and (3) the different “signaling-competent” partners of RP105 that regulates osteoclastogenesis.

## Background

Osteoclasts, multinucleated bone-resorbing cells, are required for bone remodeling. Osteoclasts originate from embryonic erythromyeloid progenitors, bone marrow hematopoietic stem cells, and mononuclear precursors, which are usually present in peripheral circulation and bone marrow (Takayanagi, 2007; McGrath et al., 2015). Studies suggest that osteoclasts are generated via the fusion of hematopoietic stem-cell-derived monocytic precursors in the presence of conducive growth factors macrophage colony-stimulating factor (MCSF/CSF-1) and receptor activator of nuclear factor- kappa B ligand (RANKL) (Udagawa et al., 1990). Excessive osteoclast activity contributes to bone loss, whereas reduced osteoclast function is associated with the development of osteopetrosis (Jacome-Galarza et al., 2019). Bacterial inflammatory diseases such as periodontitis (Blasco-Baque et al., 2017; Gu and Han, 2020) and osteomyelitis (Taubman et al., 2005), multiple myeloma (Westhrin et al., 2020), and metabolic diseases like diabetes mellitus (Watanabe et al., 2013) result in hyperactive osteoclastogenesis and progress into severe osteolytic bone diseases. Some autoimmune diseases such as systemic lupus erythematosus (SLE) (Qiao et al., 2020) and osteosarcoma (Endo-Munoz et al., 2010) induce the development of hyperostosis or arthritis as bone destruction is hampered in such cases. Recently, numerous studies have demonstrated that radioprotective 105 kDa (RP105 or CD180) and its ligand, myeloid differentiation protein 1 (MD1) are involved in inflammatory disease-induced osteoclastogenesis and bone loss.

Toll-like receptors (TLRs) play an important role in the pathophysiology of infectious diseases and inflammatory disorders (Akira et al., 2001). A total of 10 TLRs, i.e., TLR1-10 have been reported in humans. Although RP105 is a non-canonical member of the TLR family, RP105 is the most frequently encountered TLR on cells, including different kinds of bone marrow cells like bone marrow monocytes (Kikuchi et al., 2018) and immune cells including B cells (Miura et al., 1998; Ogata et al., 2000; Nagai et al., 2002; Yazawa et al., 2003), dendritic cells (DCs) (Divanovic et al., 2005) and macrophages (Liu B. et al., 2013). Multiple myeloma cells express RP105 suggesting its role in the pathophysiology of MM (Bohnhorst et al., 2006). Lipopolysaccharides (LPS) or other TLR ligands stimulate the release of proinflammatory cytokines and chemokines in immune cells such as B cells and T cells (Liu et al., 2010). Proinflammatory cytokines such as tumor necrosis factor (TNF)-α, interleukin (IL)-1, and IL-6 promote osteoclastogenesis (Ru and Wang, 2020). Osteoclast differentiation in pathological conditions is different than in physiological conditions. RP105 and MD1 are considered to be the negative regulators of TLRs, particularly TLR4 and TLR2 (Schultz and Blumenthal, 2017), and TLR-related proinflammatory cytokine production (Miura et al., 1998; Chaplin et al., 2011; Liu B. et al., 2013; Bastiaansen et al., 2014). This negative regulation might act as a compensatory mechanism to mitigate exacerbated inflammation. The binding of RP105 to MD-1 results in the activation of B cells, DCs, monocytes, and macrophages, and these RP105/MD-1-mediated phenomena enhance upon LPS stimulation (Schultz and Blumenthal, 2017). This phenomenon could be responsible for excessive osteoclastogenesis and bone loss in inflammatory diseases. Further research on the RP105/MD-1-associated signaling during osteoclastogenic differentiation of precursor cells are needed to provide insights on the development of novel strategies to mitigate inflammation and bone loss during inflammatory diseases.

## Physiological Osteoclastogenesis

**FIGURE 1:**
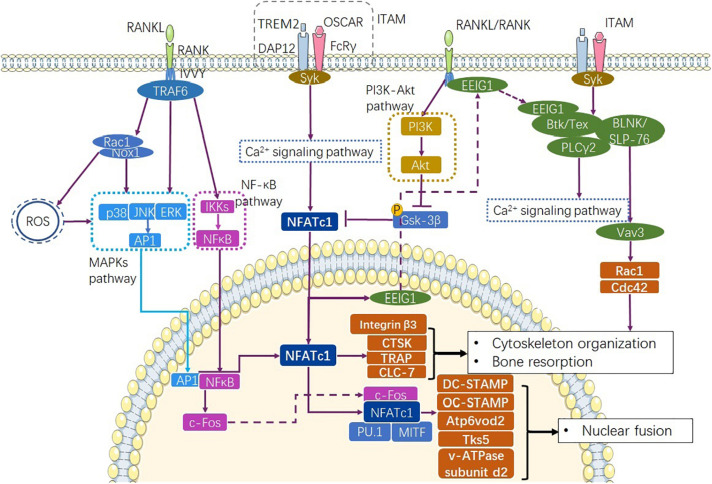
Pathways involve in physiological osteoclastogenesis. RANKL/RANK complex recruits TRAF6 to express AP1 through MAPKs and NF-κB through NF-κB signaling pathway (Gyori and Mocsai, 2020). At the same time, Ca^2+^ signaling pathway mediated by ITAM signaling promotes transcription of NFATc1 as well as the production osteoclastogenic factors CTSK, TRAP, and CLC-7. RANKL/RANK and ITAM signaling together complete the differentiation progress though the Ca^2+^ signaling pathway (Park et al., 2017). RANKL/RANK also enhance NFATc1 via inhibiting GSK-3β (Shin et al., 2014). ITAM, immunoreceptor tyrosine-based activation motif.

In normal physiological conditions, osteoclastogenesis depends on RANKL and MCSF, which are produced by osteoblast lineage cells (Ueki et al., 2007). MCSF regulates mononuclear precursor survival and proliferation, whereas RANKL regulates precursor cell fusion and maturation (Park et al., 2017). RANKL binds to the RANK receptor expressed on the surface of mononuclear cells, forms a tripolymer, and recruits TNF receptor-associated factor 6 (TRAF6) via its cytoplasmic C-terminal domain to trigger downstream signaling cascades (Takayanagi et al., 2000). TRAF6 are two important adapter proteins involved in the NF-κB and MAPK pathways (Moresco et al., 2011) (Figure 1). Expression of TRAF6 results in the production of the receptor for activated C kinase 1 (Rac1) (Kim et al., 2009) and NADPH oxidase 1 (Nox1) (Lee et al., 2005). Rac1 and Nox1 produce reactive oxygen species (ROS) and induce the phosphorylation of p38, extracellular signal-regulated kinase (ERK), and c-Jun N-terminal kinase (JNK), ultimately resulting in MAPK pathway activation and activator protein-1 (AP1) expression (Mao et al., 2006). TRAF6 activates NF-κB by inducing the nuclear localization of NF-κB via the phosphorylation of IkB kinases (IKKs) (Okamoto K. et al., 2017). As important downstream targets of the RANKL signaling pathway in the early stage of osteoclastogenesis, AP1 and NF-κB activate nuclear phosphoprotein AP-1 transcription factor comprising Fos (c-Fos) (Grigoriadis et al., 1994; Takayanagi et al., 2002). In the late stage of RANKL signaling, c-Fos cooperates with nuclear factor of activated T-cells cytoplasmic 1 (NFATc1), as well as other transcription factors, like interferon regulatory factor 4 (PU.1) and microphthalmia-associated transcription factor (MITF) to induce dendritic-cell-specific transmembrane protein (DC-STAMP), vacuolar proton pump subunit Atp6v0d2, and c-Src substrate Tks5, and regulate osteoclast fusion (Park et al., 2017).

Immunoglobulin-like receptor/immunoreceptor tyrosine- based activation motif (ITAM) signalings such as TREM-2/DAP12 and OSCAR/FcRγ activate Ca^2+^ signaling, recruit tyrosine-protein kinase SYK (Syk), and lead to the rapid activation of NFATc1 (Shinohara et al., 2008; Negishi-Koga and Takayanagi, 2009) (Figure 1). The RANKL/RANK complex also induces NFATc1 expression and nuclear localization by reducing the phosphorylation of glycogen synthase kinase-3β via the production of protein kinase C β type (PKCβ) (Shin et al., 2014) and activation of the phosphatidylinositol-3-kinase (PI3K)-Akt pathway (Moon et al., 2012) (Figure 1). As a key initiation factor of osteoclastogenesis, NFATc1 regulates the expression of integrin β3 (Crotti et al., 2006), DC-STAMP (Yagi et al., 2005), osteoclast stimulatory transmembrane protein (OC-STAMP) (Miyamoto et al., 2012), and V-type proton ATPase (v-ATPase) subunit d2 (Lee et al., 2006) via the Ca^2+^ signaling pathway. Furthermore, NFATc1 cooperates with c-Fos and Jun to induce the transactivation of tartrate-resistant acid phosphatase (TRAP) (Matsumoto et al., 2004; Matsuo et al., 2004), and promotes the transactivation of the osteoclast-associated immunoglobulin-like receptor (OSCAR) upon interacting with PU.1 and MITF (Kim K. et al., 2005; Kim Y. et al., 2005) (Figure 1). NFATc1 exhibits the phenomenon of autoregulation (Negishi-Koga and Takayanagi, 2009). At the same time, NFATc1 induces the expression of early estrogen-induced gene 1 (EEIG1), which triggers crosstalk between RANKL/RANK and the Ca^2+^ signaling pathway by regulating Btk/Tec (Choi et al., 2013). Btk/Tec interacts with B cell linker protein (BLNK)/SLP-76 and PLCγ2 to induce Ca^2+^ oscillation and calmodulin and calcineurin activation. Moreover, ITAM signaling activates Rac1 and Cdc42 production to modulate cytoskeleton organization through the Syk/SLP-76/Vav3 signaling axis (Kim et al., 2009) (Figure 1). These transcription factors are specific to osteoclasts and directly activate cell fusion and osteoclastic bone resorption.

**FIGURE 2:**
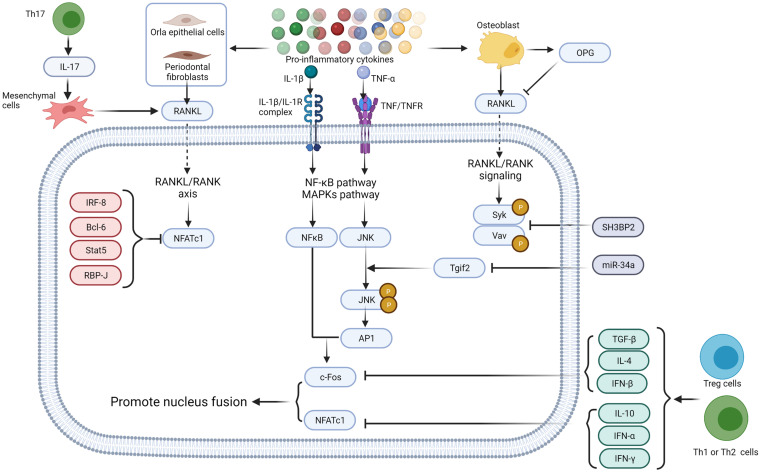
Proinflammatory cytokines regulate osteoclastogenesis via direct interaction with their receptors. Proinflammatory cytokines such as IL-1β, IL-6, IL-17, and TNF-α secreted by different immune cells induce RANKL production in endothelial cells (Batool et al., 2020), fibroblasts (Nagasawa et al., 2007; Hienz et al., 2015), and osteoblast lineage cells (Algate et al., 2016). OPG, secreted by osteoblast, works as a decoy receptor of RANKL to negatively regulate osteoclastogenesis (Lam et al., 2000). Intracellular IRF-8, Bcl6, Stat5, and RBP-J also negatively regulate osteoclastogenesis by inhibiting the expression of NFTAc1 (Ru and Wang, 2020). SH3BP2 (Ueki et al., 2007; Levaot et al., 2011) and miR-34a (Krzeszinski et al., 2014) negatively regulates Syk, Vav, and JNK protein phosphorylation. TGF-β, IFN-α, IFN-β, IFN-γ, IL-4, and IL-10 secreted by immune cells inhibit c-Fos thereby restrain osteoclastogenesis (Gao et al., 2007; Fujii et al., 2012; Xiong et al., 2016; Amarasekara et al., 2018), whereas IL-17 expressed by Th17 cells up-regulates RANKL expression in mesenchymal cells (Sato et al., 2006). Figure was created using BioRender.com.

In physiological osteoclastogenesis, osteoprotegerin (OPG) negatively regulates the RANKL-RANK signaling pathway and works as a decoy receptor for RANKL (Leite et al., 2015) (Figure 2). Several factors, such as IRF-8 (Zhao et al., 2009), Bcl6 (Miyauchi et al., 2010), Stat5 (Hirose et al., 2014), and RBP-J (Zhao et al., 2012; Li et al., 2014) are known to negatively regulate osteoclastogenesis by preventing the expression of NFTAc1. In addition, the adaptor protein SH3BP2 and microRNA such as miR-34a are reported to inhibit osteoclastogenesis (Ueki et al., 2007; Levaot et al., 2011). SH3BP3 regulates the phosphorylation of Syk and Vav whereas miR-34a influences the transforming growth factor-induced factors (Tgif2) to regulate the JNK protein phosphorylation (Krzeszinski et al., 2014). Interferon (IFN)-γ and IL-4 expressed by activated T cells, Th1 and Th2 cells, CTLA-4, TGF-β and IL-10 expressed by Treg cells inhibit osteoclast differentiation, whereas IL-17 expressed by Th17 cells upregulates RANKL expression in mesenchymal cells (Sato et al., 2006).

## Osteoclastogenesis Under Pathological Conditions

Systemic diseases such as diabetes mellitus (Blasco-Baque et al., 2017), hyperthyroidism (Baliram et al., 2012), SLE (Lim et al., 2011, 2012), rheumatoid arthritis (Murata et al., 2017; Rauber et al., 2017), and neoplastic diseases like multiple myeloma are associated with excessive osteoclastogenesis and osteoporosis (Westhrin et al., 2020). Similarly, inflammatory diseases caused by bacterial infections such as periodontitis (AlQranei and Chellaiah, 2020; Gu and Han, 2020), peri-implantitis (Yu et al., 2018), and osteomyelitis (Kavanagh et al., 2018) are associated with enhanced osteoclastogenesis leading to bone resorption. Although the pathogeneses of these conditions are different, there is consensus that the inflammatory cytokines produced in these diseases play an important role in osteoclastogenesis and bone destruction (Takayanagi, 2007).

**FIGURE 3:**
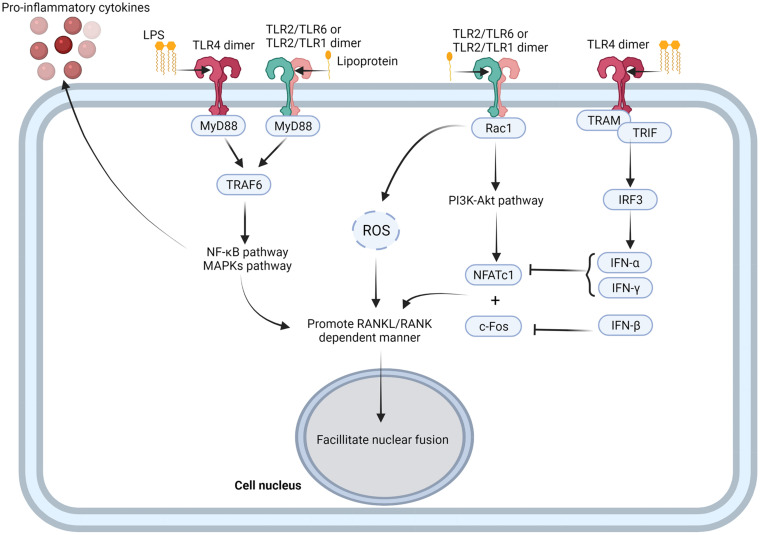
TLRs-mediated activation of osteoclastogenesis. TLR4 and TLR2 combine with PAMPs, such as LPS and lipoprotein, to form TLR4 dimer, TLR2/TLR1, and TLR2/TLR6 dimer. TLRs recruit TRAF6 with MyD88, and activate NF-κB and MAPK pathway (Souza and Lerner, 2019). NF-κB and MAPK pathways not only promote inflammatory cytokine production from osteoclast precursor cells, but also promote RNAKL/RANK dependent manner and facilitate nuclear fusion of osteoclast precursor cells (Souza and Lerner, 2019). TLR2/TLR1 dimer and TLR2/TLR6 dimer also activate PI3K-Akt pathway through recruitment of Rac1 and facilitate expression of NFATc1 (Shen et al., 2010; Kanzaki et al., 2016), while TLR4 dimer promotes expression of IFN-α and IFN-β though TRAM/TRIF/IRF3 signaling axis to negative regulate NFATc1 (Liu et al., 2016; Kwon et al., 2019). Figure was created using BioRender.com.

As typical pathogen-associated molecular patterns (PAMPs), LPS and other lipoproteins stimulate TLR4 and TLR2 (Taubman et al., 2005) to enhance complete RANKL-dependent osteoclastogenesis (AlQranei and Chellaiah, 2020). In RANKL-dependent osteoclastogenesis, LPS binds to TLR4/MD-2 complex. LPS/TLR4/MD-2 directs the recruitment of myeloid differentiation primary response protein (MyD88) via the C-terminal Toll/IL-1 receptor domain (TIR domain). TIR domain is homologous to the intracellular domain of interleukin-1 receptor of TLR4. TRAF6, recruited by MyD88, activates the NF-κB and MAPK pathways, resulting in the nuclear localization of NFκB and AP1 (Kobayashi et al., 2001; Sun et al., 2019) (Figure 3). Further, in the presence of RANKL, proinflammatory cytokines such as TNF-α and IL-1 enhance the osteoclastic phenotype of monocytes and macrophages (Liu et al., 2009) in an autocrine/paracrine manner, independent of RANKL (Nason et al., 2009) (Figure 2). TNF-α induces osteoclast formation via both the RANKL and TNF-α/TNFR axis dependent. In the presence of TNF-α, RANKL primes macrophages to differentiate into osteoclasts, and this phenomenon is completely abrogated by OPG, a decoy receptor of RANKL (Lam et al., 2000). RANKL commits the cells to the osteoclastic lineage and TNF-α ensures the induction of differentiation through TNF-α receptor (TNFR) signaling, suggesting a synergistic relationship between RANKL and TNF-α (Fuller et al., 2002). In contrast, LPS/TLR4 signaling initiates RANKL-dependent osteoclastogenesis partially by promoting RANKL production in osteoblasts (Algate et al., 2016), gingival fibroblasts (Nagasawa et al., 2007; Hienz et al., 2015), oral epithelial cells (Batool et al., 2020), and DCs (Alnaeeli et al., 2007). Inhibition of TLR4 and TLR2 in osteoblasts results in decreased RANKL expression even after exposure to LPS (Tang et al., 2011). Proinflammatory cytokines such as IL-6 and TNF-α also enhance osteoclastogenesis by promoting RANKL production in osteoblasts (Taubman et al., 2005).

Notably, LPS structure varies among microbial species (Wang et al., 2015) that may induce different signaling pathways downstream of TLRs. For example, *Porphyromonas endodontalis* LPS had been reported to enhance RANKL expression in osteoblasts via the JNK pathway (Tang et al., 2011). *Porphyromonas gingivalis* LPS enhances RANKL expression in osteoblasts via activating JNK and AP1 transcription (Burns et al., 2006). Whereas *Escherichia coli* LPS stimulates RANKL expression via ERK or PI3K signaling, without the involvement of NF-κB (Tang et al., 2011). In contrast to *E. coli* LPS, *Porphyromonas gingivalis* LPS activates TLR2 (Burns et al., 2006) and stimulates RANKL-dependent periodontal bone resorption via TLR2 signaling (Lin et al., 2014).

## TLRs During Inflammatory Bone Resorption

In general, TLRs positively regulate osteoclastogenesis as they interact with pathogenic factors or endogenous therapeutic factors and activate the inflammatory NF-κB and MAPK pathways with or without MyD88 to induce cytokine production (Moresco et al., 2011). TLR2 could form a complex with TLR1 or TLR6 to recruit Rac1 to promote production of ROS and activate PI3K-Akt signaling pathway (Shen et al., 2010; Kanzaki et al., 2016). Moreover, TLR4 activate the non-canonical MyD88-independent TRIF pathway (Souza and Lerner, 2019). Activation of the MyD88-dependent pathway mainly leads to the production of proinflammatory cytokines, whereas the TRIF pathway triggers interferon production (Souza and Lerner, 2019). Most proinflammatory cytokines like TNF-α, IL-1β, and IL-17 function as promoters of osteoclastogenesis, whereas interferons, including IFN-α, IFN-β, and IFN-γ stimulated with TLRs, like TLR4, act as inhibitors of osteoclastogenesis (Figure 2) (Amarasekara et al., 2018). Upon ligand stimulation, TLRs, especially TLR4, TLR2, TLR1, and TLR6 suppress the osteoclastogenic ability of the precursor cells via a MyD88-denpendent manner (Takami et al., 2002) (Figure 3). Several studies have reported that the addition of TLR agonist along with RANKL at the early stages of osteoclast differentiation inhibits osteoclast formation (Zhang et al., 2011; Chen et al., 2015; Kassem et al., 2015, 2016). Bone marrow macrophages (BMMs) isolated from long bones of mice and pre-treated with LPS fail to differentiate into osteoclasts with or without RANKL (Liu et al., 2009). This is possibly due to the activation of TLR4 with *E. coli* LPS, which downregulates RANKL-induced activation of NFATc1 (Liu et al., 2009). Activation of TLR2 with *Porphyromonas gingivalis* LPS also inhibits the expression of NFATc1 (Zhang et al., 2011) and c-Fos (Okamoto K. et al., 2017). This suggests the presence of various downstream signaling pathways between RANK and TLR2. Similar results had been reported in human peripheral blood monocyte cells (Takami et al., 2002) and human CD14^+^ monocyte cultures (Ji et al., 2009). Recently, LPS was also found to promote TLR4 mRNA and protein expression in MC3T3-E1 cells and to inhibit osteoblast differentiation by downregulating matrix mineralization and alkaline phosphatase activity, which play key roles in skeletal mineralization (Liu et al., 2016). Amcheslavsky et al. found that CpG-ODN, a ligand of TLR9, stimulates sustained and transient phosphorylation of ERK with or without RANKL (Amcheslavsky and Bar-Shavit, 2007). Although the duration of ERK phosphorylation directly influences the induction of c-Fos, CpG-ODN degrades c-Fos mRNA and protein to inhibit its’ induction by RANKL (Amcheslavsky and Bar-Shavit, 2007).

IFN-β induced by TLR4 signaling is an important negative regulator of RANKL-related osteoclastogenesis. IFN-β combines with the IFNAR receptor to recruit JAK1/TYK2, activate the JAK-STAT signaling pathway, and inhibit c-Fos production (Takayanagi et al., 2002). It has been reported that haptoglobin decreases osteoclast formation via activation of TLR4 and induction of IFN-β (Kwon et al., 2019). In mouse BMMs, haptoglobin also decreases osteoclast formation via TLR4 and increases mRNA and protein expression of IFN-β. Treatment of mice with haptoglobin results in a decrease in osteoclasts, even after co-stimulation by RANKL injections (Kwon et al., 2019).

## RP105: An Unconventional Members of the TLR Family

### Unique Structure of RP105 and Its’ Ligand MD-1

TLRs harbor “horseshoe”-like N-terminal leucine-rich repeat (LRR) domains as the ligand binding sites (Moresco et al., 2011). This LRR domain contains 20–30 amino acids and a high proportion of hydrophobic leucine residues (Yoon et al., 2011). The TIR domain of TLRs facilitates the recruitment of adaptor molecules and activation of conserved signaling cascades (Moresco et al., 2011). TLRs recruit MyD88 via their TIR domains, thereby leading to the activation of NF-κB, IFN regulatory factors, and transcription factors downstream of MAPKs (Moresco et al., 2011). At the protein level, the transmembrane domain and the N-terminal LRR domain of RP105 share 30% sequence homology with the TLR4 (Yoon et al., 2011; Schultz and Blumenthal, 2017). LPS, a typical PAMP widely present on the membranes of gram-negative bacteria, functions as an agonist of RP105. LPS has three functional groups, i.e., O-antigen, core oligosaccharide, and lipid A (Maldonado et al., 2016). O-antigen, also known as O polysaccharide, comprises repeating oligosaccharide units and is in direct contact with the external milieu. The O-antigen is structurally diverse and is therefore responsible for the immunological-specificity (Raetz and Whitfield, 2002). Core oligosaccharide, a structurally conservative region, links O-antigen to lipid A and maintains the integrity of the bacterial outer membrane (Maldonado et al., 2016). Lipid A is a hydrophobic moiety that anchors LPS to the outer leaflet of the outer membrane and also serves as the functional group for binding to the TLRs (especially TLR4) and activating the downstream NF-κB and MAPKs signaling pathways (Maldonado et al., 2016). In LPS-induced bone loss, lipid A regulates the expression of osteoclast differentiation factor (ODF) in primary osteoblasts and enhances the differentiation of osteoclast precursors (Kikuchi et al., 2001). In murine osteoblasts, lipid A also enhances RANKL expression and ERK activation (Kikuchi et al., 2003). The most common lipid A is the Kdo2-lipid A type which contains 4–6 acyl chains (Raetz et al., 2007). Previous studies suggest that the acylate structure directly influences LPS-induced inflammation (Herath et al., 2013). Expression of TLR4 and TLR2 differs in human gingival fibroblasts upon their stimulation with Tetra- and Penta-acylated LPS (LPS_1690_ and LPS_1435__/__1449_) isolated from a major periodontal pathogen *Porphyromonas gingivalis* (Herath et al., 2013). LPS_1435__/__1449_ fails to activate the NF-κB and p38 MAPK pathways. LPS_1435__/__1449_ has weaker stimulatory potential in the production of inflammatory cytokines GM-CSF, CXCL10, G-CSF, IL-6, IL-8, and CCL2 than that of LPS_1690_ (Herath et al., 2013). The binding of Hexa-acylated lipid A to MD-2 (the ligand of TLR4) induces a conformational change in the F126 loop of MD-2. Consequently, structural deformation is induced in TLR4, and the contact area of C-terminal TIR domains, and signal acquisition efficiency is enhanced (Scott et al., 2017). Moreover, the Penta-acylated LPS of *E. coli* is recognized as being a clinically applicable vaccine in inflammatory reactions (Ledov et al., 2019) and cancer immunotherapy (Jeong et al., 2020). Nemoto et al. compared the regulation of cementoblast function by LPS_1690_ and LPS_1435__/__1449_ in 6 h and found that RANKL production in the murine cementoblast cell line treated with LPS_1690_ is three times higher than LPS_1435__/__1449_ (Nemoto et al., 2006).

MD-1 is the ligand of RP105 and a member of the group I lipid-recognition family. MD1 shares approximately 20% sequence identity with MD-2 at the protein level (Yoon et al., 2010). MD-1 harbors a hydrophobic cavity to accommodate the lipid A moiety of LPS, which then binds to RP105 and activates downstream signaling (Nagai et al., 2002). This cavity is highly flexible and can be precisely adjusted to match its volume based on the lipid ligand acylation structure (Yoon et al., 2010). Yoon et al. suggested that large endotoxin molecules like Hexa-acylated lipids act as a “plug” to stabilize the flexible folding of MD-1 (Yoon et al., 2011). Three-dimensional simulation analysis of the molecular structure of MD-1 revealed that this cavity could expand up to seven times its original volume when bound to Hexa-acylated lipid A (Ortiz-Suarez and Bond, 2016). A recent *in vitro* study revealed that *Porphyromonas gingivalis* LPS_1690_ significantly upregulates the expression of RP105 transcripts compared to LPS_1435__/__1449_ (Ding et al., 2017). As a receptor expressed on macrophages, RP105 also mediates mycobacterial-lipoprotein-induced macrophage activation (Blumenthal et al., 2009). *Mycobacterium tuberculosis* is the key causative agent of human tuberculosis. Mycobacterial LPS lacks the teichoic acid residue present in the LPS of gram-positive and gram-negative bacteria. A recent report suggested that in lipoproteins such as the 19-kDa lipoprotein of *Mycobacterium tuberculosis*, the position of non-polar alanine residues but not the lipid moiety determines RP105 dependency (Schultz et al., 2018). This expands our understanding of the ligands of RP105 and also indicates that this receptor recognizes lipid PAMPs via numerous mechanisms. Greater attention should be paid to the relationship between RP105 and bacteria that cannot be defined by gram staining.

### Mediators in RP105-Dependent Signaling

After binding to ligands, two TLRs usually form a face-to-face “M” shaped dipolymer that brings the extracellular C-domains close to each other (Moresco et al., 2011). This conformational change allows two TIRs to form a dimer and ensures cytokine recruitment (Yoon et al., 2011). The activation of MyD88 and TRIF is TIR dimer dependent (Moresco et al., 2011). Instead of the TIR region, RP105 has a short C-domain cytoplasmic chain with 11 tyrosine residues, which does not allow RP105 to directly recruit MyD88 nor to activate MyD88-dependent downstream signaling (Kimoto et al., 2003; Ohto et al., 2011). Hence, researchers have speculated that RP105 cannot directly activate conventional TIR domain-mediated TLR signaling, and therefore, it requires “signaling-competent” partners to activate cellular responses (Kimoto et al., 2003; Schultz and Blumenthal, 2017). Based on this hypothesis, several “signaling-competent” partners of RP105 have been recognized, including CD19, Gp96, CD150, and Pim-1L.

CD19 regulates innate immunity via RP105 signaling in B lymphocytes (Miura et al., 1998; Zarember and Godowski, 2002; Yazawa et al., 2003; Nagai et al., 2013) (Table 1). Although CD19 deficiency does not affect RP105 expression, the anti-RP105 antibody induces CD19 phosphorylation in B cells. The splenic B cells from CD19^–/–^ mice express less RP105 antibody compared to B cells from wildtype mice (Yazawa et al., 2003). Anti-RP105 ligation induces normal proliferation of B cells in MyD88-deficient mice and anti-RP105 antibody and LPS significantly reduced B cell proliferation in CD19^–/–^ mice (Yazawa et al., 2003). Both anti-RP105 antibody and LPS simulates CD19 phosphorylation in splenic B cells, suggesting a central role for the RP105/CD19 signaling in B cell activation (Singh et al., 2014). Besides, anti-RP105 antibody treatment not only enhances IgG and IgM production in memory B cells, but also promotes the production of other serum antibodies (except IgGb and IgA) in mature B cells independent of memory B cells, T cells, and TLR adaptor proteins (Chaplin et al., 2011).

**TABLE 1:** Potential “signaling-competent” partners of RP105/MD-1 during osteoclastogenesis.

Cell type	Signaling-competent	Associated pathways	References
Monocytes	CD14	Ca^2+^ pathway, PI3K-Akt pathway, and crosstalk with NF-κB pathway	Zhang et al. (2019)
Bone marrow macrophages	TLR2	Akt-PI3K and NF-κB pathway	Yu et al. (2015)
	Pim-1L	JAK/STAT and PI3K-Akt pathway	Hu et al. (2009); Kim et al. (2010)
	TLR7, TLR9	Ca^2+^ pathway	Zhang et al. (2019)
CD19 + B lymphocytes	CD19	Ca^2+^ pathway; Akt-PI3K pathway; MAPKs pathway (MyD88-independent signaling); NF-κB pathway (MyD88-independent signaling)	Yazawa et al. (2003); Hebeis et al. (2005); Shinohara et al. (2008); Yoon et al. (2009); You et al. (2017); Gavali et al. (2019)

Gp96, an endoplasmic reticulum chaperone, plays an important role in the B cell secretory pathway. Absence of Gp96 decreases cell surface expression of RP105 and MD-1, suggesting the requirement of Gp96 for the assembly of the RP105/MD-1 complex (Weekes et al., 2012). This complex is different from TLR4/MD-2, as the absence of Gp96 does not influence TLR4 and MD-2 expression (Staron et al., 2010). Recently, the interaction between CD150 and RP105 has been shown to regulate Akt and MAPK signaling in chronic lymphocytic leukemia B cells *in vitro* and *in vivo* (Gordiienko et al., 2017). CD150 and RP105 cross-linking in chronic lymphocytic leukemia B cells leads to the activation of Akt and MAPK networks. More importantly, the co-ligation between these two receptors reduces the phosphorylation of Akt, ERK1/2, and c-Jun, and completely blocks p38-MAPK phosphorylation (Gordiienko et al., 2017). Serine/threonine kinase proviral integration site for Moloney murine leukemia virus 1 (Pim-1) has a long (Pim-1L) and short (Pim-1S) isoforms (Tursynbay et al., 2016). Egli et al. found that Pim-1L colocalizes and physically associates with RP105 via its N-terminal extension. This complex has autonomous signaling capabilities in transporting inflammatory signals into B cell survival programs (Egli et al., 2015). RP105/Pim-1L triggers the upregulation of anti-apoptotic BAD protein phosphorylation in a Pim-1 kinase-dependent manner in mature B cells (Egli et al., 2015). Moreover, in human primary B cells, monoclonal antibody-stimulated cross-linking of RP105 present a new pathway for cell survival, proliferation, and activity of B cells (Egli et al., 2015).

## RP105 and Pro-Inflammatory Cytokines

### TLR4

Although the RP105 signaling pathway depends on TLR4, TLR2, and TLR9, the RP105 is being increasingly considered as a negative regulator of downstream signaling pathways of these receptors, including NF-κB, MAPKs, and Akt-PI3K in monocytes and immune cells (Park et al., 2017). RP105 acts as a physiological and endogenous inhibitor of TLR4 in macrophages (Yu et al., 2015; Carreras-Gonzalez et al., 2018), DCs (Gorczynski et al., 2006; Tada et al., 2008; Okamoto N. et al., 2017), and monocytes (Honda et al., 2007; Wezel et al., 2015; Zhang et al., 2019). RP105 negatively regulates TLR4-mediated IFN-β expression (Okamoto N. et al., 2017), signaling elements such as p38MAPK (Kikuchi et al., 2018), kinase phosphorylation levels of c-Jun/AP1 (Dong et al., 2019) and Akt (Yu et al., 2015), and production of proinflammatory cytokines such as IL-6 (Wezel et al., 2016), IL-1β (Chen et al., 2019), and TNF-α (Louwe et al., 2014; Carreras-Gonzalez et al., 2018). Targeted inhibition of MD-1 stimulates the TLR4/MyD88/NF-κB signaling axis in colitis-induced mice (Chen et al., 2019), which also proves the role of RP105 as a TLR4 inhibitor.

Ohto et al. analyzed the structure of the RP105/MD-1 complex combined with the TLR4 dimer and generated a hypothetical docking model of the hetero-oligomer consisting of TLR4/MD-2 and RP105/MD-1 (Ohto et al., 2011). In this model, the RP105/MD-1 dimer inserts itself into the TLR4/MD-2 dimer, with the C-termini of RP105 extending outward. This docking model confirmed that RP105 interacts with the TLR4/MD-2 complex and is accessible to the C-termini of TLR4. This model also revealed the mechanism of RP105/MD-1-mediated inhibition of TLR4 signaling. RP105/MD-1 blocks the association of the cytoplasmic TIR domains between two TLR4 monomers and inserts into the homodimerization interface of the TLR4/MD-2/LPS complex to inhibit LPS-induced TLR4/MD-2 oligomerization. RP105/MD-1 and TLR4 accommodate lipid molecules in proximity to the lipopeptide-binding site of TLR4, suggesting that RP105/MD-1 facilitates downstream signaling by transferring the ligand to TLR4. Yoon et al. (2011) also propose two hypothetical models, with the first one being similar to the model suggested by Ohto et al. (2011). The second model proposes a cross binding structure located on the descending side of the N-terminal domain in RP105. This model explains why the RP105/MD-1 complex usually forms a unique 2:2 stoichiometry rather than a 1:1 organization observed in the TLR4/MD-2 inhibition complex, i.e., RP105/MD-1 inserts into the homodimerization interface of TLR4/MD-2/LPS to close the entrance sites of LPS on the MD-2 cavity. Yoon et al. supported the credibility of the first model. Ortiz-Suarez et al. compared the structures of the bovine MD-1 monomer protein and the bovine and human RP105/MD-1 complexes (Ortiz-Suarez and Bond, 2016). Their findings confirmed the malleability and stability of MD-1. With these characteristics, MD-1 binds to bulky endotoxins like LPS and forms an expansive structure. Therefore, the RP105/MD-1/LPS complex can competitively block the TLR4/MD-2 oligomerization interface. Their results support the hypothesis that RP105 acts as a “decoy receptor” and “sink” to sequester LPS from TLR4 under certain conditions.

### TLR2

TLR2 activation does not depend on the extracellular segment helper proteins. X-ray crystallography confirmed that TLR2 forms heterodimers with TLR1 (Jin et al., 2007) or TLR6 (Kang et al., 2009) to receive ligands like lipoproteins and lipopeptides. Nagai et al. reported that RP105^–/–^ B cells exhibit low sensitivity to lipid A and lipopeptides Pam3CSK4 and MALP-2 (Nagai et al., 2005). Furthermore, RP105^–/–^ mice displayed impaired polyclonal antibody production, especially IgG2b, IgG3, and IgM, upon stimulation with LPS and Pam3CSK4 (Nagai et al., 2005). Liu et al. emphasized that RP105-dependent reduction of polyclonal antibody production in B cells and deficiency of RP105 in BMMs does not impair lipid A or lipopeptides-induced TNF-α production (Liu B. et al., 2013). Moreover, the response to synthetic TLR2 agonists such as Pam3CSK4 and MALP-2 in RP105^–/–^ macrophages and DCs is similar to that of wildtype cells (Murofushi et al., 2015). This cell-specific difference suggests different roles of RP105 in mediating activation of different immune cells.

RP105 also regulates the NF-κB pathway via TLR2 and TLR4 in HEK cells stimulated with *Lactobacillus plantarum* N14, neutral, and acidic exopolysaccharides (Murofushi et al., 2015). Furthermore, in epithelial cells, RP105/MD-1 is involved in the recognition of phosphopolysaccharides produced by lactic acid bacteria. RP105/MD-1 interacts with phosphopolysaccharides to activate the NF-κB and PI3K pathways and produces proinflammatory cytokines in intestinal epithelial cells (Laiño et al., 2016).

### TLR7 and TLR9

The ligands of TLR7 and TLR9 are endogenous RNA and DNA, respectively, which are implicated in autoimmune diseases such as SLE (Yang et al., 2018a). The ligands of TLR7 and TLR9 restrain RP105 expression in human peripheral blood monocytes and immune cells including macrophages and DCs (You et al., 2017). Knocking out RP105 in these cells downregulates the expression of TLR7 and TLR9. An *in vivo* study showed that the proportion of RP105-negative B cells and DCs is significantly increased in patients with SLE and MRL/lpr mice (mutant mice susceptible to lupus) (Yang et al., 2018a). This degenerative feedback is based on the Lyn-SHP-1/2 axis (Yang et al., 2018a). Lyn inhibits B cell activation in SLE via inhibiting the TLR-MyD88 axis (Liossis et al., 2001; Flores-Borja et al., 2005; Ban et al., 2016). These reports suggest a negative feedback mechanism of TLR pathway activation, i.e., RP105 negatively regulates the activation of TLR7 and TLR9-mediated pathways, and TLR7 and TLR9-mediated pathways also act as negative regulators of RP105 activation.

## RP105 Involved in Bone Metabolism During Inflammatory Diseases

### Multiple Myeloma

Multiple myeloma is a heterogeneous bone marrow cancer characterized by increased osteoclast formation and activity. More than 90% of multiple myeloma patients have extensive lytic bone destruction (Coleman et al., 2020). Kikuchi et al. reported that in almost all skeletal-related events, the RP105/MD-1 complex is expressed on multiple myeloma cells but not on normal counterparts, and the complex abundance is markedly up-regulated under adherent and hypoxic conditions (Kikuchi et al., 2018). They also found that LPS and anti-RP105 antibodies, but not other TLR ligands, enhances the growth of multiple myeloma cells via activation of MAP kinases ERK and JNK (Kikuchi et al., 2018). Additionally, directly inoculated multiple myeloma cells attach to bone marrow stromal cells in a murine xenograft model. LPS stimulation significantly increased the number of RP105/CD138 double-positive cells (Kikuchi et al., 2018). Knockdown of RP105 canceled the LPS response of multiple myeloma cells *in vitro* and *in vivo* (Kikuchi et al., 2018). Via promoter analysis, Furukawa et al. identified IKZF1 (Ikaros) as a pivotal transcriptional activator of the RP105 gene. The transcription of RP105 on multiple myeloma cells is also activated by increasing Ikaros expression and its binding to the promoter region (Furukawa et al., 2018). Furukawa et al. performed pharmacological targeting of Ikaros with lenalidomide to improve the response of multiple myeloma cells to LPS in an RP105-dependent manner *in vitro* and *in vivo* (Furukawa et al., 2018). Recently, Kikuchi et al. found that the administration of lenalidomide prevented the LPS-triggered activation of multiple myeloma cells by targeting RP105 (Kikuchi and Furukawa, 2020). These studies suggest that the RP105/MD-1 pathway may represent a novel mechanism of growth regulation of multiple myeloma cells in a bone marrow milieu.

### Osteosarcoma

Compared to normal bone, the expression of osteoclastogenesis markers and antigen presentation is reduced in osteosarcoma (Endo-Munoz et al., 2010). Chen et al. compared the RNA-seq data of 82 osteosarcoma samples as well as their clinical information and constructed a prognostic model (Chen et al., 2020). They identified five predictors, including RP105, MYC, PROSER2, DNA11, and FATE1 are the optimal multivariable Cox regression model. In accordance with the Cox regression analyses, RP105 was downregulated and the other four genes were upregulated in the high-risk group. Among the five prognostic genes, only RP105 leads NF-κB activation and negatively correlates with osteosarcoma survival. Chen et al. proposed that a high expression of RP105 might indicate a high anticancer activity of lymphocytes and suppress the growth of osteosarcoma tumor (Chen et al., 2020).

### Diabetes Mellitus

Diabetes-induced osteoporosis is commonly encountered in clinics. In the insulin resistance state, macrophages are activated by the recognition of free fatty acids from hypertrophied adipocytes or LPS through the TLR4/MD-2 complex to induce TNF-α production (Watanabe et al., 2012, 2013). Paracrine and/or autocrine TNF-α combines with TNFR on preosteoclasts. In continuous exposure to TNF-α, preosteoclasts differentiate into fully mature osteoclasts and this event is independent of RANKL (Blasco-Baque et al., 2017; Coleman et al., 2020).

Yasuharu et al. reported that RP105 mRNA is highly expressed in most adipose tissue macrophages (Watanabe et al., 2012). A high-fat diet (HFD) remarkably increases RP105/MD-1 complex expression on the M1 subset of adipose tissue macrophages. Both RP105 and TLR4 are involved in HFD-induced NF-κB activation in the epididymal white adipose tissue of mice. RP105 KO and MD-1 KO mice had lesser HFD-induced adipose tissue inflammation and insulin resistance compared to TLR4 KO and wildtype mice (Watanabe et al., 2012). RP105/MD-1 complexes contribute to HFD-induced obesity, adipose tissue inflammation, and insulin resistance independent TLR4 signaling (Nagai et al., 2013). Liang et al. isolated monocytes from human peripheral blood mononuclear cells and found that exposed lipid infusion *in vivo* enhances the LPS-stimulated IL-1β secretion in monocytes (Liang et al., 2018). IL-1β increases the level of TLR4 protein as well as phosphorylation of JNK and p38/MAPK in monocytes, which directly correlates with reduced peripheral insulin sensitivity (Liang et al., 2018). Additionally, increased lipid levels are linked to RP105, as well as multiple genes associated with osteoclastogenesis such as MAP3K7 and CXCL10 (Liang et al., 2018).

### SLE

SLE is a chronic autoimmune inflammatory disease. Bone erosions and inflammatory bone loss are common features in autoimmune arthritis such as in rheumatoid arthritis (RA) (Lim et al., 2011). However, bone erosions and inflammatory bone loss are usually absent in patients with SLE and are observed in < 5% of cases (Lim et al., 2012). Qiao et al. found that the deposition of IgG, monocytes/macrophages, and TNF-α is required for the development of RA in SLE (Qiao et al., 2020). However, lupus serum or IgG inhibits RANKL-induced differentiation of monocytes into osteoclasts in a dose-dependent manner, as lupus IgG competes for FcγRI binding with RANKL (Qiao et al., 2020).

The number of RP105-negative B cells is significantly increased in peripheral blood mononuclear cells from patients with SLE and is positively associated with disease severity (Koarada et al., 1999; Koarada and Tada, 2012). Only RP105-negative B cells obtained from patients with SLE spontaneously produced IgG and IgM *in vitro* (Kikuchi et al., 2002). These studies suggest that RP105-negative B cells may be responsible for the production of autoantibodies. Moreover, the binding of RP105 by anti-RP105 antibodies could inhibit the IFN-α-induced interferon stimulated gene (ISG) expression in B cells both *in vitro* and *in vivo* (You et al., 2017). IFN-α inhibits the fusion of osteoclast precursor cells by downregulating c-Fos expression in a dose-dependent manner (Xiong et al., 2016; Amarasekara et al., 2018). Mechanistically, RP105 down-regulates the tyrosine phosphorylation of signal transducer and activator of transcription 2 (STAT2) induced by IFN-α via a Lyn-dependent pathway (You et al., 2017). Moreover, SLE is characterized by the formation of a variety of autoantibodies by hyper-reactive B cells. B-cell antigen receptors recognize endogenous DNA and RNA-associated auto-antigens and deliver to endosomal TLR9 and TLR7, respectively (Yang et al., 2018a). In this way, overactivation of B cells leads to the excess production of autoantibodies, contributing to the inflammatory amplification loop characteristic of SLE. Although TLR7 and TLR9 signaling could downregulate RP105 expression and attenuate the inhibitory effect of anti-RP105 on the activation of IFN-α signaling in B cells (You et al., 2017), the critical role of RP105 in regulating TLR7 and TLR9-mediated activation of macrophages and DCs suggests that RP105 could be a potential therapeutic target for the treatment of SLE (Yang et al., 2018a).

### Rheumatoid Arthritis

Rheumatoid arthritis is an inflammatory joint disease, which is characterized by chronic inflammations that irreversibly affect the surrounding bone tissue (Gyõri and Mócsai, 2016). As a chronic autoimmune disease, the inflammatory condition in rheumatoid arthritis not only enhances bone degradation by raising the crosstalk between osteoclastogenesis and the immune system, but also stimulates TLR2 on osteoblasts and increases the production of RANKL (Kassem et al., 2015). Moreover, TLR7 induces RANKL expression and increases osteoclasts derived from peripheral blood CD14 + monocytes in rheumatoid arthritis (Kim et al., 2019). Because of the ability of RP105 in blocking TLR2 and TLR7, it can be speculated that RP105 could regulate osteoclastogenesis in rheumatoid arthritis via a TLR2/TLR7. Although there is no evidence directly proving the influence of RP105 on osteoclastogenesis in rheumatoid arthritis, an intersection of positional and functional candidate information analysis provided some evidence of novel rheumatoid arthritis loci near the PI3KR1/RP105 gene (Kim et al., 2019). Another study demonstrated an antibody against mouse CD79b, which blocks B cell proliferation, is induced via RP105 but not via TLR4 and TLR. Anti-CD79 reduces inflammation and improves synovial hyperplasia as well as bone and cartilage destruction (Kim et al., 2019). These results suggest that RP105 could be a biomarker in predicting the occurrence and development of rheumatoid arthritis. Furthermore, blocking RP105 may be a promising treatment for preventing bone resorption in rheumatoid arthritis.

## RP105 and Osteoclastogenesis

In the following sections, we describe RP105 as a regulator in activating monocytes, macrophages, and B cells, as well as summarize osteoclastogenesis regulating signaling pathways that are activated by RP105 in different osteoclast precursor cell types.

### Monocyte-Derived Osteoclasts

The mononuclear precursors located in peripheral blood and the bone marrow are the key source of osteoclast precursors (Vaananen, 2005). Monocytes derived from the spleen and thymus also differentiate into osteoclasts under a suitable microenvironment (Udagawa et al., 1990). In hind limb ischemia (HLI) mouse model, before HLI development, the predominant resident monocyte subtype was LY6C^low^, accounting for 30% of the total CD11b^+^ cells in the gastrocnemius and adductor muscles of both WT and RP105^–/–^ mice (Bastiaansen et al., 2014). After the development of HLI, RP105 knockout leads to increased activation of Ly6C^high^ monocytes, implying that RP105 deficiency inhibits the regenerative response of mice following ischemia (Bastiaansen et al., 2014). RP105 also directly influences the migratory capacity of monocytes. Wezel et al. compared the monocytes influx and neutrophil influx to the peritoneum of low-density lipoprotein receptor (LDLr) deficient (LDLr^–/–^) mice and LDLr/RP105 double knockout (LDLr^–/–^/RP105^–/–^) mice fed with Western-type diet (Wezel et al., 2015). They found although the neutrophil influx was unaltered between the two kinds of mutant mice, the monocytes influx was almost 3-fold lower in LDLr^–/–^/RP105^–/–^ mice than LDLr^–/–^ mice (Wezel et al., 2015). More important, the results of *in vitro* experiment showed a down-regulation of CCR2 in the two kinks of receptor knockout monocytes when stimulated with LPS, which is more pronounced in LDLr^–/–^/RP105^–/–^ monocytes than LDLr^–/–^ monocytes (Wezel et al., 2015). CCR2 is an important chemokine receptor of classical monocytes. Deficiency or low expression of CCR2 severely inhibited the osteoclastogenesis potential of monocytes (Puchner et al., 2018).

**FIGURE 4:**
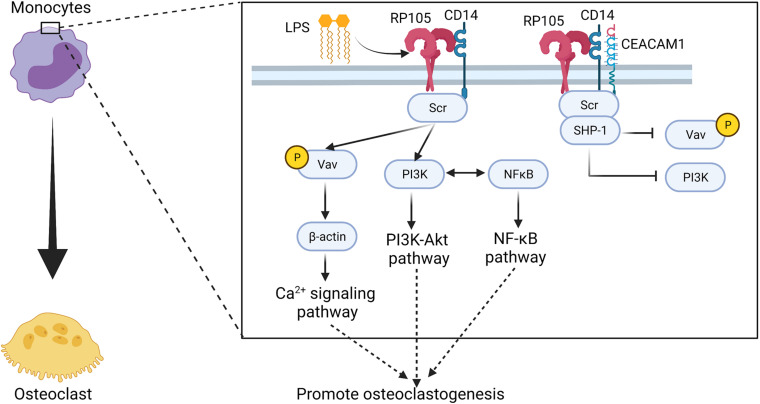
Potential regulatory manner of RP105 in driving osteoclastogenesis of monocytes. RP105 may promote the differentiation of monocytes into osteoclasts via a RP105/MD-1/CD14/Src/Vav1/β-actin axis (Zhang et al., 2019). At the same time, with the stimulation of LPS, RP105/MD-1/CD14/Scr also activates the PI3K-Akt pathway and induces crosstalk with the NF-κB signaling pathway (Zhang et al., 2019). But, in presence of CEACAM1, SHP-1 recruitment blocks the tyrosine phosphorylation of Vav1, and sequestrates pVav and β-actin from the downstream signaling axis of RP105 (Zhang et al., 2019). Figure was created using BioRender.com.

RP105, MD-1, and CD14 form a complex on murine bone monocytes in response to the early phase expression of IL-6 in LPS-treated mice (Zhang et al., 2019). Upon *Listeria monocytogenes* infection in mice, IL-6 production is also influenced by carcinoembryonic antigen-related cell adhesion molecule-1 (CEACAM1) via the G-CSFR-STAT3 pathway (Pan and Shively, 2010). CEACAM1 regulates IL-1β in LPS-treated neutrophils via the TLR4-Syk pathway (Lu et al., 2012). In these two events, CEACAM1 is recruited by an activated receptor such as G-CSFR or TLR4 phosphorylated by Src. CEACAM1 recruits and dephosphorylates SHP-1 to activate the insulin receptor in the liver (Poy et al., 2002), and recruits epidermal growth factor receptor in the epithelial cells (Abou-Rjaily et al., 2004) or B cell receptors (Lobo et al., 2009; Chen et al., 2011). However, neither TLR4 mRNA nor protein was detected in murine monocytes, which means that the murine bone marrow monocytes do not express TLR4 (Ketloy et al., 2008). Instead, RP105/MD-1/CD14/Src/PI3K signaling occurs in response to LPS stimulation, which also crosstalks with NF-κB to regulate the production of IL-6 (Zhang et al., 2019) (Figure 4 and Table 1). At the same time, with the presence of CEACAM1, the activation of RP105 also leads to recruitment of SHP-1 and prevents PI3K activation, inhibit the tyrosine phosphorylation of Vav1, and sequestrate pVav and β-actin from the RP105 complex (Zhang et al., 2019).

### Macrophage-Derived Osteoclasts

Macrophage and osteoclast share a common monocyte precursor (Nason et al., 2009). BMMs and monocyte macrophages are the key sources of osteoclast precursors (Boyle et al., 2003). Mature macrophages and osteoclasts two competing differentiation outcomes from myeloid progenitors share many common antigens (AlQranei and Chellaiah, 2020). Because of the differential role of macrophages and osteoclasts in bone remodeling, the balance between these two types of cells is especially important for bone metabolism. Broadly, activated macrophages are divided into two major types, M1 and M2 macrophages. M1 macrophages are proinflammatory and are classically activated by LPS or T helper 1 (Th1) cell-related cytokines such as IFN-γ (Chang et al., 2008; Sinder et al., 2015). M2 macrophages are identified as anti-inflammatory macrophages activated by Th2 cell-related cytokines such as IL-4 and IL-13 (Chang et al., 2008; Sinder et al., 2015). M1 macrophage-related cytokines TNF-α, IL-6, and IL-1β induce osteoclastogenesis. While the M2 macrophage-related cytokines IL-4 and IL-10 inhibit osteoclastogenesis through the inhibition of NFATc1 (Varol et al., 2015). Thus, the polarization of macrophages (M1/M2) is important for the determination of osteoclastogenesis (Yang and Wan, 2019).

Under inflammatory conditions, the recruitment and proliferation rate of monocytes (Wezel et al., 2015, 2016) and macrophages (Wezel et al., 2016) in RP105^–/–^ mice is higher than in WT mice. Several studies support the concept that RP105 also initiates the polarization of macrophages into M1 types (Nagai et al., 2013). The mRNA expression and protein levels of RP105 and MD-1 in macrophages are up-regulated when in contact with tumor cells (Watanabe et al., 2012). Czimmerer et al. analyzed that IL-4 as well as IFN-γ + IFN-α stimulate human monocyte-derived macrophages and identified RP105 as well as MS4A4A, SLA, and ENPP2 as novel IL-4 regulated alternative activation markers (Czimmerer et al., 2012). *Mycobacterium tuberculosis* lipopeptide ligands stimulate RP105 on macrophages and induce RP105-dependent TNF-α and IL-6 production by macrophages. Moreover, di- and tripalmitoylated variants of this lipopeptide can elicit an equivalent RP105-dependent response, indicating that the lipid moiety is required for macrophage activation but this event is RP105-independent (Schultz et al., 2018). RP105 is a key determinant of macrophage activation in *Mycobacterium tuberculosis* infection. Myeloid-derived suppressor cells (MDSCs) play an immunosuppressive role in the pathogenesis of inflammatory diseases. Compared with non-treated controls, the expression of RP105 of MDSCs, especially granulocytic MDSCs (G-MDSCs), from mice challenged with LPS was significantly increased (Dong et al., 2019). *In vitro*, binding of RP105 with an anti-RP105 antibody not only inhibits the expansion of MDSCs by preventing the phosphorylation of signal transducer and activator of transcription 3 (STAT3) but also reduces the immunosuppressive activity of MDSCs on M1 macrophage polarization through the inhibition of Arg-1 expression (Dong et al., 2019). The injection of anti-RP105 antibody significantly aggravates pathological lesions in mice stimulated with LPS (Dong et al., 2019). Furthermore, the injection of anti-RP105 antibody inhibits the accumulation of G-MDSCs in LPS-challenged mice and reduced the immunosuppressive activity of G-MDSCs on M1 macrophage polarization (Dong et al., 2019).

**FIGURE 5:**
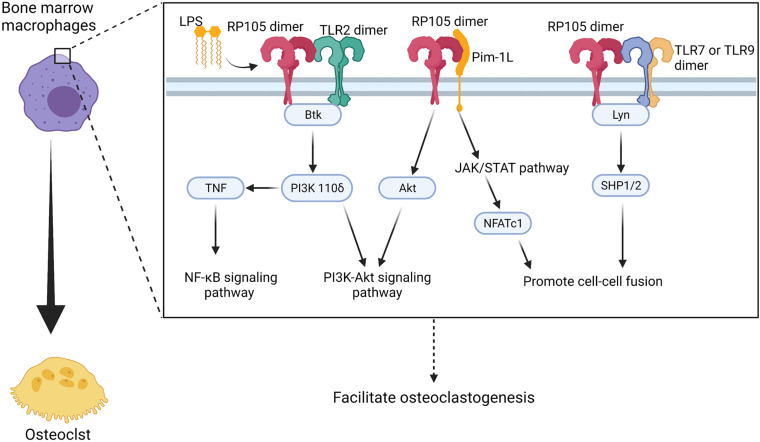
Potential regulatory manner of RP105 in activating osteoclastogenesis of bone marrow macrophages. RP105 promotes the differentiation of bone marrow macrophages into osteoclasts with different “signaling-competent” partners. As a result, there’re three potentially regulate manner of RP105 in the differentiation process: (1) TLR2 works as a “signaling-competent” of RP105 (Carreras-Gonzalez et al., 2018) and forms RP105/TLR2/Btk axis to up-regulate PI3K p110δ to activate the PI3K-Akt as well as promotes production of TNF and activation of NF-κB pathways (Blumenthal et al., 2009; Yu et al., 2015); (2) Pim-1L works another “signaling-competent” of RP105 (Egli et al., 2015) and stimulate expression of NFATc1 through JAK/STAT pathway (Kim et al., 2010) and activate Akt to promote PI3K-Akt signaling pathway (Hu et al., 2009); (3) RP105 also could inhibit TLR7 and TLR9 signaling and the downstream Lyn/SHP-1/2 manner, which activate Ca^2+^ signaling pathway and promote cell-cell fusion (Yang et al., 2018a). Figure was created using BioRender.com.

Carreras-González et al. reported increase in CD14 protein and gene expression but decrease in RP105 expression in *Borrelia burgdorferi* stimulated BMMs (Carreras-Gonzalez et al., 2018). *In vitro* differentiated human macrophages also show reduced expression of RP105. However, in this study, human blood monocytes show downregulation of CD14 and upregulation of RP105. Carreras-González et al. also observed that silencing of RP105 significantly reduces TNF-α and IL6 production in mouse mononuclear macrophage cell line RAW264.7 (Carreras-Gonzalez et al., 2018). PI3K is a classical multifunctional signaling protein, which influences several cellular metabolic processes such as autophagy and differentiation (Werner et al., 2010). PI3K consists of one regulatory subunit and one of four p110 catalytic subunits, including class 1A: α, β, δ, and class 1Bγ (Vanhaesebroeck et al., 2005). The p110δ subunit of PI3K is the central regulator in inducing inflammatory responses in immune cells (Kim et al., 2007; Werner et al., 2010). In macrophages, PI3K p110δ is required for the fission of TNF-α containing vesicles and their transport to the cell surface upon stimulation of LPS (Low et al., 2010). In mycobacteria-infected macrophages, RP105 and Btk are required for PI3K p110δ activation (Blumenthal et al., 2009; Yu et al., 2015) (Figure 5 and Table 1). Moreover, PI3K signaling in macrophages induced by bacterial infection contribute to the production of cytokines TNF, IL-6, G-CSF, and CCL5 that contribute to macrophage recruitment and polarization (Andrade et al., 2009). In myocardial ischemia and reperfusion injury, RP105 activates the PI3K-Akt signaling pathway, which protects patients from ischemia/reperfusion-induced cardiac injury and myocardial pyroptosis (Guo et al., 2021).

Notably, PI3K recruitment is dependent on the YXXM motifs (Wang et al., 2002; Troutman et al., 2012). CD19 has two YXXM motifs in the cytoplasmic region, which means that CD19 recruits the p85α sites of PI3K (Wang et al., 2002). However, instead of the TIR domain, RP105 contains at least one tyrosine residue in the cytoplasmic tail, which cannot be embedded into a classical YXXM motif. As a result, RP105 can either directly stimulate PI3K signaling or activate this pathway with a competent partner (Schultz and Blumenthal, 2017) such as CD19 (Aiba et al., 2008). However, CD19 is usually detected on lymphocytes circulating in peripheral blood (Wang et al., 2002), and B cells in the spleen (Wang et al., 2002), bone marrow (Engel et al., 1995), thymus, and lymph nodes (Ishiura et al., 2010). To date, there are no reports of the presence of CD19 on macrophages. Therefore, the YXXM-containing partner of RP105 remains to be elucidated in macrophages. There is a possibility that the tyrosine tail of RP105 phosphorylates in response to an activating interaction, thereby provides a recruitment platform for Btk or PI3K regulatory subunits in macrophages (Yu et al., 2015). TLR2 and TLR3 respond to the mycobacteria-induced activation of PI3K-Akt signaling pathway in macrophages (Lasunskaia et al., 2006; Liu H. et al., 2013; Bai et al., 2014). In the Staphylococcus aureus infection rat model, RP105 activation in macrophages induces the accumulation of TLR2 and increased TLR2-associated inflammatory cytokines production via a MyD88 axis (Blumenthal et al., 2009). Some researchers conjectured that RP105-dependent activation of the PI3K-Akt signaling pathway might require TLR2 (Yu et al., 2015; Huang et al., 2020). But it worth noticing, the activation of PI3K via the TLR2-associated axis provides crosstalk with MAPKs and NF-κB pathway. Interestingly, the phosphorylation of p38, JNK, p65, and IκB is not altered in RP105 deficiency macrophages during mycobacterial infection (Blumenthal et al., 2009; Yu et al., 2015). However, RP105 deficiency and RP105/TLR2 deficiency in macrophages directly reduced the production of TNF and IL-6 (Yu et al., 2015; Schultz et al., 2018). Based on the available literature, TLR2 is still considered as the most possible RP105 signaling partner in the activation of the PI3K-Akt signaling pathway in macrophages.

Pim-1L, another “signaling-competent” partner of RP105, contains an additional proline-rich motif at the N-terminus. Thus, it can interact with proteins and crosstalk with signaling cytokines other than Pim-1S (Tursynbay et al., 2016). Pim-1 positively regulates Akt phosphorylation and activates apoptosis in human and mouse hematopoietic malignancies and prostate cancer (Hu et al., 2009) (Figure 5). More importantly, Pim-1 regulates NFATc1 in osteoclastogenesis via the JAK/STAT pathway (Kim et al., 2010). However, whether Pim-1L exhibits a potential ability to link RP105 and the JAK/STAT pathway and directly regulate osteoclastogenesis remains to be elucidated.

TLR7 expression is upregulated in synovial tissues (Roelofs et al., 2005), fibroblasts (Carrión et al., 2011), macrophages (Alzabin et al., 2012), and DCs (Roelofs et al., 2005). Recently, TLR7 was determined to regulate osteoclastogenesis in rheumatoid arthritis via RANKL expression in synovial fibroblasts (Kim et al., 2019). At the same time, RP105 negatively regulates TLR7 and TLR9-mediated activation of macrophages and DCs via Lyn/SHP-1/2 signaling (Yang et al., 2018a), suggesting a possible mechanism of osteoclastogenesis in patients with rheumatoid arthritis.

### B Cell-Derived Osteoclasts

**FIGURE 6:**
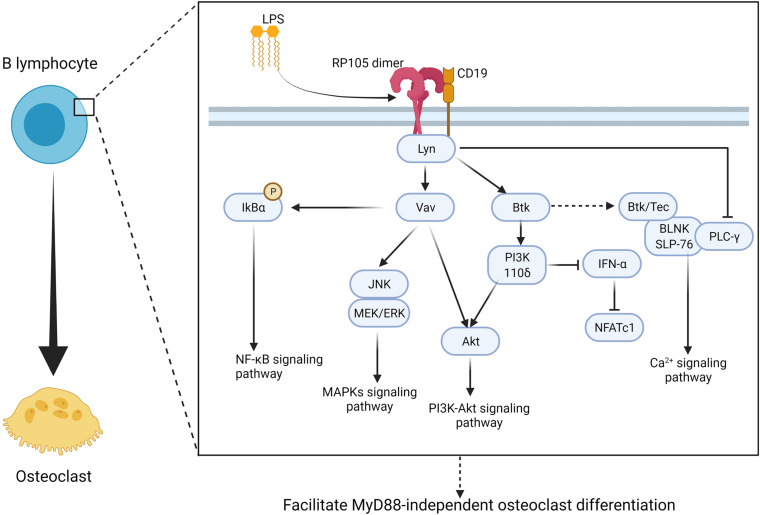
RP105 activates MyD88-independent manner of CD19 + B lymphocyte and promote CD19^+^ B lymphocyte to derive into osteoclast. RP105/CD19 initiates Lyn activation and phosphorylation then promotes MyD88-independent osteoclast different manner of CD19^+^ B lymphocyte via two major pathways: (1) promote IκBα to promote NF-κB signaling (Yazawa et al., 2003; Hebeis et al., 2005), activate JNK, MEK/ERK to promote MAPKs signaling pathway (Chan et al., 1998; Yazawa et al., 2003; Hebeis et al., 2005), as well as activate Akt to promote PI3K-Akt signaling pathway though a Vav-associated manner (Yu et al., 2015); (2) activate PI3K 110δ to inhibit IFN-α and promote PI3K-Akt signaling pathway through Btk-associated manner (You et al., 2017). At the same time, Btk also directly promote osteoclastogenesis though Ca^2+^ signaling pathway, however, Lyn also inhibit PLCγ-mediated Ca^2+^ signaling (Shinohara et al., 2008; Choi et al., 2013). As a result, the regulate signaling axis of osteoclastogenesis in CD19^+^ B lymphocyte remain to be further discussed. Figure was created using BioRender.com.

A study showed that circulating myeloma B-lymphocytes have the potential to differentiate into osteoclast-like phenotype (Calvani et al., 2004). Researchers not only detected RANK on CD19^+^ B lymphocytes (Atkins et al., 2006) but also observed that peripheral CD19^+^ B lymphocytes isolated from patients with multiple myeloma exhibit phenotypic and functional properties of osteoclasts upon stimulation with RANKL (Calvani et al., 2004). Thus, CD19^+^ B lymphocytes are RANKL-dependent osteoclast progenitors. As discussed above, CD19 is one of the most recognized accessory proteins of RP105. The frequency of RP105^+^ cells is higher in the CD19^+^ non-switched B cell subset, and these B cells show the strongest activation in response to anti-RP105 antibody treatment (Singh et al., 2014). Moreover, phosphorylation of CD19 is completely independent of TLR4 (Yazawa et al., 2003), which suggests that RP105 may directly drive B cells to differentiate into osteoclasts via the CD19 pathway. In B cells, the interaction between RP105 and CD19 forms a complex that directly recruits Lyn kinase (Yazawa et al., 2003). This RP105/CD19 complex is also required for optimal Lyn activation and phosphorylation (Yazawa et al., 2003) (Table 1). The interaction of RP105 and LPS induces Lyn activation and CD19 phosphorylation (Yazawa et al., 2003). Although Lyn inhibits osteoclast differentiation, survival, and function by interfering with PLCγ-mediated Ca^2+^ signaling, which leads to down-regulation of osteoclastogenesis (Yoon et al., 2009; Gavali et al., 2019), it also mediates the activation of Vav (Yazawa et al., 2003). Vav protein is required for RP105 function as it regulates LPS-mediated activation of Akt, ERK, phosphorylation of IκBα (Yazawa et al., 2003; Hebeis et al., 2005). Vav1/2-deficient B cells fail to respond to LPS or anti-RP105 antibodies *in vitro* (Yazawa et al., 2003; Hebeis et al., 2005). The CD19/Lyn/Vav signaling acts upstream of MAPKs to induce JNK and MEK/ERK axis activation (Chan et al., 1998; Yazawa et al., 2003; Hebeis et al., 2005) (Figure 6). In parallel, anti-RP105 cross-linking induces ERK as well as JNK phosphorylation within a short period (Chan et al., 1998). Phosphorylation of JNK by RP105 depends on CD19, however, phosphorylation of ERK is independent of CD19 (Yazawa et al., 2003).

Besides RP105/CD19/Lyn/Vav signaling axis, RP105/CD19/Lyn also acts upstream of Btk and links RP105-PI3K signaling (Yu et al., 2015) (Figure 6). RP105/CD19/Lyn signaling plays an important role in regulating IFN-α activation in B cells. You et al. observed that the RP105 ligation inhibits IFN-α-induced tyrosine phosphorylation of signal transducer and STAT2 via RP105/CD19/Lyn (You et al., 2017). Besides, Btk also acts as a downstream effector of RP105 in PI3K activation and promotes TNF-α release in BMMs in response to mycobacterial infection (Yu et al., 2015). At the same time, Btk directly participates in osteoclast formation. It has been demonstrated that Btk and Tec kinases form a complex with the BLNK adaptor and activate an essential calcium-signaling pathway with PLCγ (Shinohara et al., 2008; Choi et al., 2013). In summary, based on the aforementioned facts, RP105 seems to promote B cells’ osteoclastic differentiation via MAPKs, PI3K-Akt, and Ca^2+^ signaling pathways (Figure 6).

### Alternative Sources of Osteoclastogenesis-Regulation

RP105 not only influences the osteoclastogenic potential of monocytes, macrophages, and other cells, but also affects the microenvironment of osteoclast differentiation by regulating the secretion of various factors by surrounding cells. RP105 deficient mice have higher CCL2 secretion in vein graft lesions than WT mice, which increases the macrophage content in these lesions (Wezel et al., 2016). *In vitro* analysis also shown that RP105^–/–^ smooth muscle cells produced a higher level of CCL2 than the control group (Wezel et al., 2016). CCL2 regulates hormonal and mechanical stimuli-induced physiological bone remodeling, as well as cancer-induced bone resorption (Siddiqui and Partridge, 2017). Besides, in a hind limb ischemia model, arteriogenesis is reduced in RP105^–/–^ mice than WT mice (Bastiaansen et al., 2014). This effect might regulate bone healing, since angiogenesis is crucial for bone repair.

Activation of RP105 also stimulates Bax, caspase-3, and cytochrome c in myocardial cells during myocardial ischemia-reperfusion injury and upregulates Bc1-2 to restrain the apoptotic process (Yang et al., 2015). When challenged with an HFD, MD-1 expression is downregulated *in vivo* and *in vitro* (Shen et al., 2019). MD-1 deficiency causes cardiac hypertrophy and fibrosis via activation of MAPK and NF-κB that accelerates myocardial injury (Shen et al., 2019). Overexpression of MD-1 alleviates the effects of HFD-induced cardiac remodeling (Shen et al., 2019). Peng et al. reported that MD-1 deletion activates the TLR4/CaMKII signaling pathway and influences the expression of Ca^2+^ handling proteins, thereby increasing the vulnerability of HFD-fed mice to ventricular arrhythmia (Peng et al., 2017). The authors hypothesized that MD-1 deletion increases susceptibility to atrial fibrillation in heart failure with presented ejection fraction (HFpEJ) by enhancing the activation of TLR4/CaMKII signaling. Compared with wildtype mice, MD-1 knockout mice challenged with aldosterone infusion downregulate Ca^2+^-ATPase 2a (SERCA2a) expression and phospholamban phosphorylation at Thr17 (Shuai et al., 2020). In the MD-1 knockout mice, sodium/calcium exchanger 1 and phosphorylation of ryanodine receptor 2 expression resulted in increased myocardial fibrosis and inflammation, proving that MD-1 regulates the activation of the TLR4/CaMKII signaling *in vivo* and *in vitro* (Shuai et al., 2020).

Qin et al. transfected mRNA-141-3p mimic to H9c2 rat cardiomyoblasts to analyze the role of miR-141-3p and RP105 in myocardial ischemia. The inhibition of miRNA-141-3p activates the RP105-dependent PI3K-Akt pathway in cardiomyocytes exposed to hypoxia. miRNA-141-3p inhibition reduces cell death, apoptosis, and generation of ROS in hypoxic H9c2 rat cardiomyocytes *in vitro*. miRNA-141-3p targets RP105 by directly binding to its 3’-UTR. Blocking RP105 reverses the effects of miRNA-141-3p inhibition in rat cardiomyoblast cells (Qin et al., 2019). The association between RP105 and miRNA-327 in myocardial ischemia has also been reported. miRNA-327 downregulates RP105 in a 3’-UTR-dependent manner. The reduction of miRNA-327 indirectly downregulates the TLR4 and TLR2 dependent-MyD88 and NF-κB signaling axis, whereas it upregulates RP105 (Yang et al., 2018b). This effect results in the reduction of myocardial infarct size, attenuates cardiomyocyte destruction, and alleviates inflammation.

## Conclusion

Despite several years of research, the differentiation progress and induction mechanism of osteoclast received wide attention. Bone marrow-derived cells and immune cells activated by TLRs have the potential to generate osteoclast. Moreover, the pro-inflammatory cytokines produced by these cells create a suitable microenvironment for osteoclastogenesis. Bone resorption and osteoporosis in cancer, diabetes mellitus, rheumatoid arthritis, and SLE are mainly caused by osteoclast overactivity. Patients with these diseases are in chronic inflammatory state or inflammatory stress state for a long time. In this state, patients have a high risk of orthopedic disease even without bacterial infection.

As a special cell member receptor, RP105 play a dual role in pro-inflammatory response. At one hand, RP105 serves as a low-affinity receptor of LPS but shares certain features with TLR4. With the simulation of LPS or other agonist, RP105 activates monocytes, macrophages, immune cells, and other peripheral tissue cells to produce pro-inflammatory cytokines, which facilitate the osteoclastogenesis. At the other hand, RP105 also negatively regulates other TLRs and reduces the production of pro-inflammatory cytokines, which seems to inhibit osteoclastogenesis to some extent. But the physiological function of RP105/MD-1 is not limited to inflammatory response. RP105 also regulates the multiple myeloma cells’ growth and attachment to bone marrow stromal cells in the murine xenograft model (Kikuchi et al., 2018). Moreover, it has been verified *in vitro* and *in vivo*, that RP105/MD-1 plays important role in the regulation of monocytes migration, which influences early atherosclerotic plaque development (Wezel et al., 2015). RP105 and MD-1 directly influence the arteriogenesis after ischemia (Bastiaansen et al., 2014), aggravates vein graft disease (Wezel et al., 2016). These effects might regulate the bone niche or mechanical stimuli-induced physiological bone remodeling. Considered with the central role and high expression of RP105/MD-1 in adipose macrophages (Watanabe et al., 2012), myocardial cells (Shuai et al., 2020), cardiomyoblast cells (Qin et al., 2019), and smooth muscle cells (Wezel et al., 2016), this complex had been suggested as a possible therapeutic target in reducing chronic inflammatory of diabetes mellitus (Watanabe et al., 2012, 2013; Nagai et al., 2013), rheumatoid arthritis (Bruhl et al., 2015), and SLE (Yang et al., 2018a).

So far, several studies have focused on how RP105 negatively regulates other TLRs, however, its signaling pathways remain inadequately explored. Although “signaling-competent” partners and downstream signaling mechanisms of RP105 remain elusive, the available evidence allows us to hypothesize that the RP105/MD-1 complex activates Ca^2+^, MAPK, NF-κB, and PI3K-Akt signaling to directly promote osteoclastogenesis of bone marrow-derived monocyte/macrophage precursor cells and a subset of B lymphocytes. Identification and characterization of RP105/MD-1 ligands will not only help in integrating the molecular mechanism of osteoclastogenesis derived from different cell sources but also help to delineate inflammation regulatory roles of RP105 under inflammatory conditions. Further insights into the biology of RP105 may inspire novel strategies to control and cure inflammation and inflammation-induced osteoclastogenesis.

## Author Contributions

JP and LG designed this study and edited the final draft. ZF wrote the manuscript. All authors approved the final version of the manuscript.

## Conflict of Interest

The authors declare that the research was conducted in the absence of any commercial or financial relationships that could be construed as a potential conflict of interest.

## Publisher’s Note

All claims expressed in this article are solely those of the authors and do not necessarily represent those of their affiliated organizations, or those of the publisher, the editors and the reviewers. Any product that may be evaluated in this article, or claim that may be made by its manufacturer, is not guaranteed or endorsed by the publisher.

## Abbreviations

AP1: activator protein-1
BLNK: B cell linker protein
BMMs: Bone marrow macrophages
CEACAM1: carcinoembryonic antigen-related cell adhesion molecule-1
DC: dendritic cell
ERK: extracellular-signal-regulated kinase
g-MDSCs: granulocytic MDSCs
IFN: interferon
Ikaros: identified IKZF1
ITAM: immunoglobulin-like receptor/immunoreceptor tyrosine-based activation motif
LPS: lipopolysaccharides
LRR: leucine-rich repeat
M-CSF: macrophage colony-stimulating factor
MD1: myeloid differentiation protein 1
MDSC: myeloid-derived suppressor cell
NFATc1: nuclear factor of activated T-cells cytoplasmic 1
OPG: osteoprotegerin
OS: osteosarcoma
PAMP: pathogen-associated molecular pattern
PI3K: phosphatidylinositol-3-kinase
Pim-1: proviral integration site for Moloney murine leukemia virus 1
Pim-1L: long isoform of Pim-1
Pim-1S: short isoform of Pim-1
PRR: pattern recognition receptor
RANKL: receptor activator of NF-kB ligand
RP105/CD180: radioprotective 105 kDa
SLE: systemic lupus erythematosus
STAT: signal transducer and activator of transcription
TIR: intracellular interleukin-1 receptor
TLR: Toll-like receptor
TNF: tumor necrosis factor
TNFR: TNF- α receptor.
